# Effects of Epigenetic Modification of PGC-1α by a Chemical Chaperon on Mitochondria Biogenesis and Visual Function in Retinitis Pigmentosa

**DOI:** 10.3390/cells11091497

**Published:** 2022-04-29

**Authors:** Yoko Ozawa, Eriko Toda, Kohei Homma, Hideto Osada, Norihiro Nagai, Kazuo Tsubota, Hideyuki Okano

**Affiliations:** 1 Department of Ophthalmology, St. Luke’s International Hospital, 9-1 Akashi-cho, Chuo-ku, Tokyo 104-8560, Japan; nagai@a5.keio.jp; 2 Laboratory of Retinal Cell Biology, St. Luke’s International University, 9-1 Akashi-cho, Chuo-ku, Tokyo 104-8560, Japan; 3 Laboratory of Retinal Cell Biology, Department of Ophthalmology, Keio University School of Medicine, 35 Shinanomachi, Shinjuku-ku, Tokyo 160-8582, Japan; riko.4267@gmail.com (E.T.); hommak@keio.jp (K.H.); 49hidet00sada@gmail.com (H.O.); 4 Department of Ophthalmology, Keio University School of Medicine, 35 Shinanomachi, Shinjuku-ku, Tokyo 160-8582, Japan; tsubota@tsubota-lab.com; 5 Department of Physiology, Keio University School of Medicine, 35 Shinanomachi, Shinjuku-ku, Tokyo 160-8582, Japan; hidokano@keio.jp

**Keywords:** retinitis pigmentosa, rhodopsin, ER stress, PGC-1α, mitochondria

## Abstract

Retinitis pigmentosa (RP) is a hereditary blinding disease characterized by gradual photoreceptor death, which lacks a definitive treatment. Here, we demonstrated the effect of 4-phenylbutyric acid (PBA), a chemical chaperon that can suppress endoplasmic reticulum (ER) stress, in P23H mutant rhodopsin knock-in RP models. In the RP models, constant PBA treatment led to the retention of a greater number of photoreceptors, preserving the inner segment (IS), a mitochondrial- and ER-rich part of the photoreceptors. Electroretinography showed that PBA treatment preserved photoreceptor function. At the early point, ER-associated degradation markers, *xbp1s*, *vcp*, and *derl1*, mitochondrial kinetic-related markers, *fis1*, *lc3*, and *mfn1* and *mfn2*, as well as key mitochondrial regulators, *pgc-1α* and *tfam*, were upregulated in the retina of the models treated with PBA. In vitro analyses showed that PBA upregulated *pgc-1α* and *tfam* transcription, leading to an increase in the mitochondrial membrane potential, cytochrome c oxidase activity, and ATP levels. Histone acetylation of the *PGC-1α* promoter was increased by PBA, indicating that PBA affected the mitochondrial condition through epigenetic changes. Our findings constituted proof of concept for the treatment of ER stress-related RP using PBA and revealed PBA’s neuroprotective effects, paving the way for its future clinical application.

## 1. Introduction

Recent progress in medical science has led to the development of new therapeutic approaches in various fields; this is also applicable for blinding diseases such as age-related macular degeneration and diabetic retinopathy [1]. However, there are currently no definitive treatments for retinitis pigmentosa (RP), a hereditary blinding disease. RP is a leading cause of blindness with a prevalence of 1:4000 [2,3], affecting more than 1.5 million persons worldwide [3]. While the associated gene mutations are congenital, visual disability progresses with age in patients with RP, and the aging society will lead to increasing numbers of affected patients, rendering RP an urgent social issue.

Rhodopsin is an evolutionarily conserved, seven-transmembrane protein, specifically produced in the rod photoreceptor cells, and acts as a visual pigment; rhodopsin mutations cause RP [4]. Among them, the P23H rhodopsin, which has a substitution of proline to histidine at position 23, is the most common cause of human autosomal dominant RP in the USA [5]. The P23H mutant rhodopsin protein has been classified as a Class II mutant causing protein misfolding, leading to disordered protein trafficking in the endoplasmic reticulum (ER) and ER stress [6,7]. More recently, autosomal dominant RPs caused by rhodopsin mutations are classified into four clusters, and P23H mutation is involved in cluster two [8].

Under ER-stress conditions, the unfolded protein response is triggered to promote adaptive alteration requiring energy expenditure. The signaling induces folding enzymes and chaperones, i.e., components of the ER-associated degradation (ERAD) machinery, to clear unfolded proteins and restore ER homeostasis. However, when ER stress exceeds the capacity of the adaptive actions, cellular function deteriorates, often leading to cell death, inducing C/EBP homologous protein (CHOP). Thus, several therapeutic approaches have been proposed for treating ER stress-related RP. Using induced pluripotent stem cells derived from a patient with RP with a rhodopsin mutation, we previously reported that rapamycin and other ER-stress inhibitors could attenuate rod photoreceptor death [7], although the in vivo effect was obscure. Others have reported that gene administration of binding immunoglobulin protein, BiP, an ER-localized chaperone that acts as a sensor of ER stress, improved photoreceptor survival and visual function in P23H mutant animals [9]. However, gene therapy cannot cover the whole area of the retina and could be invasive.

Here, we focused on 4-phenylbutyric acid (PBA), a chemical chaperon that can suppress ER stress [10,11]. PBA is a Food and Drug Administration-approved drug used to treat urea-cycle disorders, as it also acts as an ammonia scavenger [11,12]. The mechanisms of PBA action on ER-stress regulation are reported to involve its action as a chaperon, which increases the solubility of the abnormal protein [11,13,14], and/or as a transcriptional regulator of molecules involved in the ER quality control system [14]. Alternatively, its action as a histone deacetylase (HDAC) inhibitor may stabilize the transcriptional activity of spliced XBP1 [15], a key regulator of ER stress and ERAD, leading to recovery from stress [16,17]. In fact, our previous report showed that ER-stress markers were suppressed by PBA, resulting in photoreceptor protection in the retina of an acute photo-stress model [18]. However, PBA could have various roles as a chaperon [11], and the current study was designed to show the probable roles and effects of PBA in RP treatment.

In this study, we treated human P23H knock-in mice exhibiting progressive rod degeneration with daily PBA administration to show PBA’s neuroprotective effects and probable mechanisms and propel the future clinical application of PBA for retinal neuroprotection. This minimally invasive approach that uses drugs to slow degeneration and visual disability could be applicable to a large number of patients with RP in the future.

## 2. Materials and Methods

### 2.1. Animals

The P23H rhodopsin knock-in mice backcrossed to C57B6J background (provided by Dr. Palczewski, Case-Western University, Cleveland, OH, USA) [19,20] were maintained under a 12-h light/dark cycle (lights on from 8 a.m. to 8 p.m.) with free access to food and water, in an air-conditioned room (22 °C) at the animal facility of Keio University School of Medicine. Male animals were used for the study and intraperitoneally treated either with sodium phenylbutyric acid (PBA, SML0309, Sigma-Aldrich, St. Louis, MO, USA) at 10 mg/kg body weight (BW) or with phosphate-buffered saline (PBS) as a vehicle, five times a week from 2 weeks of age. All animal experiments were conducted in accordance with the ARVO Statement for the Use of Animals in Ophthalmic and Vision Research and the guidelines of the Animal Care Committee of Keio University (Approval number; 09203).

### 2.2. Histological Analyses

Mouse eyes were enucleated and fixed in 4% paraformaldehyde. Paraffin (Sakura Finetek, Tokyo, Japan) sections (8 μm), including the optic nerve head to the most peripheral region of the superior and inferior retina, were deparaffinized, followed by staining with hematoxylin and eosin. Sections were examined under a microscope equipped with a digital camera (Olympus Co., Tokyo, Japan). For immunohistochemical analyses, sections were incubated with anti-Tom20 (Santa Cruz Biotechnology, Santa Cruz, CA, USA; #sc-11415) at 1:500 and anti-rhodopsin (Thermo Fisher Scientific, Waltham, MA, USA; #MA1-722) at 1:10,000 antibodies; signals were obtained using Alexa 488-conjugated goat anti-mouse and Alexa 555-conjugated goat anti-rabbit IgGs, respectively. Nuclei were stained with Cellstain-4’,6-diamidino-2-phenylindole solution (Dojindo Molecular Technologies, Kumamoto, Japan, 2 μg/mL). Fluorescent images were obtained using a confocal microscope (TCS-SP5; Leica, Tokyo, Japan). The number of cells in the outer nuclear layer (ONL), the photoreceptor layer, and quantification of inner segment (IS) areas in a 50 μm length of the retina was evaluated at each point using ImageJ software (National Institutes of Health, Bethesda, MD, USA; available at http://rsb.info.nih.gov/ij/index.html (accessed 27 September 2021) and averaged as described previously [21,22,23,24]; data at 200 μm distance from the optic nerve in the superior retina were shown.

### 2.3. Real-Time Reverse Transcription-Polymerase Chain Reaction (RT-PCR)

Total RNA in the neural retina was isolated using TRIzol reagent (Life Technologies, Carlsbad, CA, USA) and reverse transcribed by the SuperScript VILO master mix (Life Technologies). Real-time PCR was performed using the StepOnePlus™ PCR system (Applied Biosystems, Foster City, CA, USA), and gene expression levels were quantified by the 2^−ΔΔCT^ method and normalized to the expression of Gapdh. Primers are listed in Supplementary Table S1.

### 2.4. Electroretinography (ERG) Recordings

Mice were dark-adapted for at least 12 h before conducting the ERGs modifying the methods previously described [21,25,26,27]. Briefly, mice were anesthetized with intraperitoneal combined anesthetics [midazolam 4 mg/kg of BW (Sandoz Japan, Tokyo, Japan), medetomidine 0.75 mg/kg BW (Nippon Zenyaku Kogyo Co., Ltd., Fukushima, Japan), butorphanol tartrate 5 mg/kg BW (Meiji Seika Pharma Co., Ltd., Tokyo, Japan)] and maintained on a heating pad under dim-red illumination throughout the experiment. Mouse pupils were dilated by a single eye drop of a mixture of tropicamide and phenylephrine (0.5% each; Mydrin-P^®^; Santen, Osaka, Japan). The respective ground and reference electrodes were placed on the tail and in the mouth, and the active gold wire electrodes were on the cornea. Recordings were acquired using a PowerLab System 2/25 (AD Instruments, Bella Vista, New South Wales, Australia). Each response was differentially amplified and filtered through a digital bandpass filter ranging from 0.3 to 1000 Hz. Each stimulus was delivered using a commercial stimulator (Ganzfeld System SG-2002; LKC Technologies, Inc., Gaithersburg, MD, USA). Full-field scotopic ERGs were recorded in response to a flash stimulus at intensities ranging from −2.1 to 2.9 log cd s/m^2^. Photopic ERGs were recorded after 10 min of light adaptation at flash stimuli ranging from 0.4 to 1.4 log cd s/m^2^ with a background of 30 cd s/m^2^. The a-wave amplitude was measured from the baseline to the trough, and the b-wave amplitude was from the trough of the a-wave to the peak of the b-wave. The implicit times of the a and b waves were measured from the onset of the stimulus to the peak of each wave. The automatically indicated peak points by the system were confirmed by the examiner.

### 2.5. Cell Culture

HEK293 cells (EC85120602−G0, European Collection of Authenticated Cell Cultures, Public Health England) were maintained in Dulbecco’s modified Eagle’s medium (#08459-35; Nacalai tesque, Kyoto, Japan) supplemented with 10% fetal bovine serum (Life Technologies), 100 unit/mL penicillin, and 100 μg/mL streptomycin (Nacalai tesque) at 37 °C in a humidified atmosphere of 5% CO_2_. For passaging, cells were dissociated with 0.25% trypsin/EDTA (Thermo Fisher Scientific, Waltham, MA, USA). 

### 2.6. Mitochondrial Membrane Potential Measurement 

HEK293 cells were plated on glass-based dishes (Non-coat 35 mm glass-bottom dish, D11530H, Matsunami Glass, Osaka, Japan) a day before the experiment. Cells were treated with or without 2.5 mM PBA in the culture medium for 24 h. On the day of the experiment, cells were stained with tetramethylrhodamine ethyl ester (TMRE, Thermo Fisher Scientific, Waltham, MA, USA; #T669) at 10 μM. Fluorescent images were obtained every 2 s using a fluorescent microscope (BZ-X710, KEYENCE, Osaka, Japan) with a TRITC filter (OP-87764, KEYENCE). During the imaging, (2-fluorophenyl) 6-[(2-fluorophenyl) amino] (1,2,5-oxadiazolo[3,4-e]pyrazin-5-yl) amine (BAM15, SML1760, Sigma-Aldrich, St. Louis, MO, USA) was added to the recording dish at 10 μM.

### 2.7. Cytochrome c Oxidase (CcO) Activity Measurement

HEK293 cells were treated with or without 2.5 mM PBA in the culture medium for 24 h. Then, the treated cells were incubated in the buffer in the kit before measuring the CcO activity using the Complex IV Rodent Enzyme Activity Microplate Assay Kit (Abcam, Cambridge, UK) according to the manufacturer’s instructions and our previous work [21]. The luminescent signals were measured using the Cytation 5 system (BioTek, Winooski, VT, USA).

### 2.8. ATP Measurement

HEK293 cells were treated with or without 2.5 mM PBA in the culture medium for 24 h. After treatment, cells 

S2 were recovered from the culture dish with TE-saturated phenol (NIPPON GENE Co., Ltd., Tokyo, Japan). The ATP levels in the samples were measured using the ATP Bioluminescence Assay Kit CLSII (Sigma-Aldrich, St. Louis, MO, USA) according to the manufacturer’s instructions and our previous work [21]. The luminescent signals were measured using the Cytation 5 system (BioTek, Winooski, VT, USA).

### 2.9. Chromatin Immunoprecipitation Quantitative Real-Time PCR (ChIP-qPCR)

HEK293 cells were treated with or without 2.5 mM PBA in the culture medium for 24 h. After the treatment, cells were fixed with 16% formaldehyde (28906, Thermo Fisher Scientific, Waltham, MA, USA) for 15 min at room temperature. Fixed and fractionated nuclei from HEK293 cells were resuspended in ChIP buffer from ChIP-IT High Sensitivity Kit (Active Motif cat #53040) and sonicated in microtube AFA Fiber Pre-slit Snap-Cap set on Covaris S2 instrument (Covaris, Woburn, MA, USA) at 5% duty cycle, the intensity of 4, and 200 cycles per burst for 16 min. For the immunoprecipitation, ChIP-IT High Sensitivity Kit (#53040, Active Motif, Inc., Carlsbad, CA, USA) and histone H3K27ac antibody (#39133, Active Motif) was used according to the manufacturer’s instructions. After the purification of DNA, the precipitated fraction of DNA was quantified by qPCR by using Fast SYBR Green Master Mix (#4385614, Thermo Fisher Scientific, Waltham, MA, USA) and StepOnePlus™ PCR system (Applied Biosystems). The fold-change of % inputs at the probes to that at GAPDH promoter in each sample was quantified by Ct values of the probes. Primers of the probes are listed in Supplementary Table S2.

### 2.10. Statistical Analysis

All results are expressed as the mean ± standard deviation. The values were processed for statistical analyses using one-way analysis of variance with Tukey’s post hoc tests for comparisons among three groups or two-tailed Student’s *t*-tests for comparisons between two groups using SPSS Statistics 24 (IBM, Armonk, Hamlet, NY, USA). Differences were considered statistically significant at *p* < 0.05.

## 3. Results

### 3.1. PBA Promoted Photoreceptor Survival in P23H Knock-In Heterozygotes (P23H RP Models)

**Figure 1:**
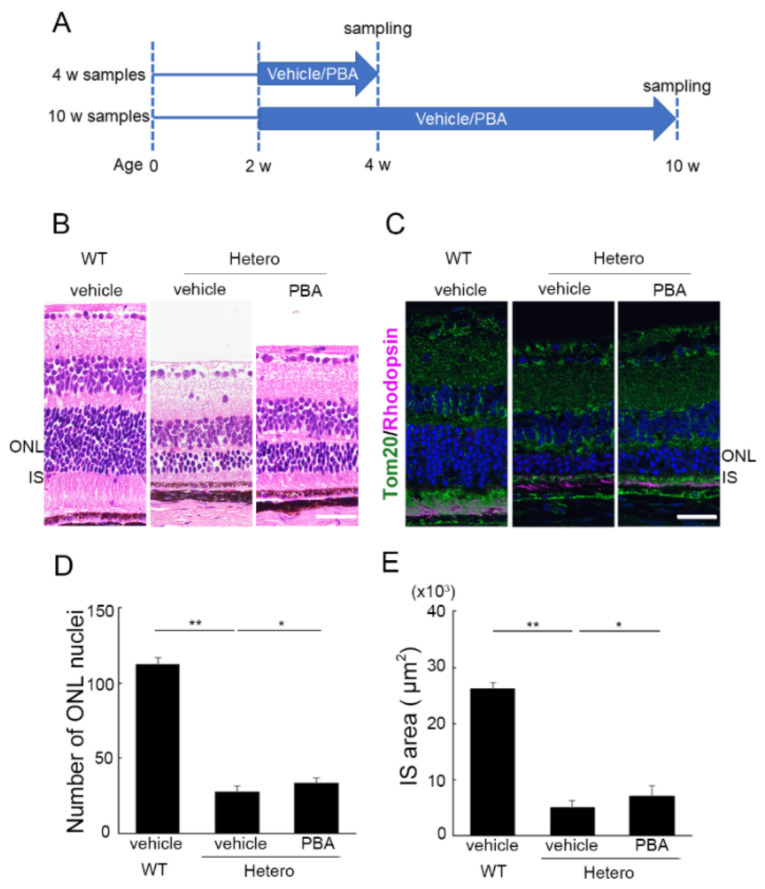
**Photoreceptor survival by PBA in the P23H knock-in heterozygotes (P23H RP models)** (**A**) P23H knock-in heterozygotes (P23H RP models) started daily treatment with PBA five times a week from 2 weeks of age until the time point of analyses. (**B**–**E**) Hematoxylin-eosin staining and immunohistochemistry showed that a greater number of photoreceptors (**B**–**D**) (rhodopsin in pink), and area of IS marked by mitochondria marker, Tom20 (green) (**C**,**E**) were reduced in the P23H RP models compared with wild-type mice at 10 weeks of age; however, the levels were significantly higher in the models treated with PBA at the same time point. (**D**,**E**) Data at 200 μm distance from the optic nerve in the superior retina. RP, retinitis pigmentosa; IS, inner segment. Data are shown as mean ± standard deviation. *n* for WT treated with vehicle, 3; P23H RP models treated with vehicle, 8–9; P23H treated with PBA, 8. ONL, outer nuclear layer; IS, inner segments. * *p* < 0.05, ** *p* < 0.01. Scale bar, 20 μm.

A previous report showed that the thickness of the photoreceptor layer does not change in P23H knock-in heterozygous retinitis pigmentosa models (P23H RP models), compared with wild-type, until postnatal day 12 and that degeneration gradually progresses thereafter [19]. Thus, continuous administration of either PBA or the control vehicle was started at the age of 2 weeks in the current study (Figure 1A). Consequently, the remaining photoreceptors, as assessed by the number of photoreceptor nuclei, were significantly greater at the age of 10 weeks in the retinas of P23H RP models treated with PBA than in those treated with vehicle; at that time, photoreceptor loss was significant in the P23H RP models (Figure 1B–D). The remaining area of the photoreceptor IS, where mitochondria, labeled with Tom20 and ER, are densely distributed, was also greater in the PBA-treated mice (Figure 1B,C,E). These results indicated that PBA promoted photoreceptor survival and suppressed IS degeneration in the P23H RP models. The effects of PBA were significantly observed in the superior part of the retina (Figure S1A,B).

### 3.2. PBA Preserved Visual Function in the P23H RP Models

**Figure 2:**
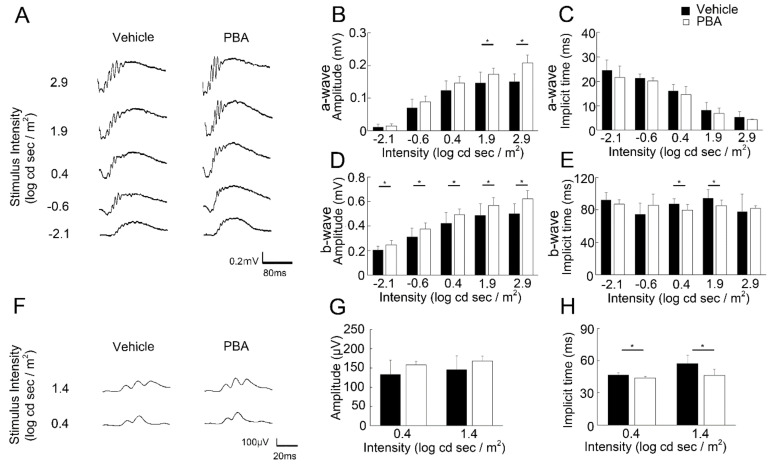
**Preservation of visual function by PBA in the P23H RP models** (**A**–**H**) ERG recorded at 10 weeks of age. (**A**) Representative wave forms of scotopic ERG from individual mice at a stimulus intensity from −2.1 to 2.9 log cd s/m^2^ in the P23H RP models with or without PBA treatment. Amplitudes of a-waves representing rod photoreceptor function (**B**) and amplitudes (**D**) and implicit times (**E**) of b-waves representing subsequent retinal cell function showed that PBA played a vision-protective role in rod photoreceptors. Representative wave forms of photopic ERG from individual mice at a stimulus intensity of 0.4 and 1.4 log cd s/m^2^ (**F**) showed that implicit time of b-wave representing cone photoreceptor function was preserved by PBA treatment (**H**). ERG, electroretinography. Data are shown as mean ± standard deviation. (**A**–**E**) *n* = 10–11. (**F**–**H**) *n* = 5–6. * *p* < 0.05.

Impairment of rod photoreceptor function measured with scotopic ERG is already found at the age of 6 weeks, and cone function measured with photopic ERG is found at 10 weeks, both followed by a gradual progress in the P23H RP models as previously reported [20]. Scotopic (Figure 2A–E) and photopic (Figure 2F–H) ERGs were measured to examine whether histological rescue by PBA treatment would contribute to the retention of visual function. In the scotopic ERG, a-wave amplitudes reflecting rod photoreceptor function were greater, and the respective b-wave amplitudes and implicit times reflecting subsequent retinal neural function to photoreceptors were greater and shorter in the PBA-treated P23H RP models than in vehicle-treated models at the age of 10 weeks, indicating that the function of the rod system was partly retained by continuous PBA treatment. Respective greater amplitudes of a and b waves after PBA treatment had most likely reflected the number of survived rod photoreceptors, and shorter implicit time of b-wave may have resulted from the better synaptic function of the remaining rod system. In b-wave photopic ERG, which mainly shows cone system function, the implicit time was shorter after treatment with PBA, indicating that the cone system was also partly protected by PBA treatment in the P23H RP models.

### 3.3. The Protective Effect of PBA Was Detected in the Photoreceptors of P23H RP Models before Photoreceptor Loss

**Figure 3:**
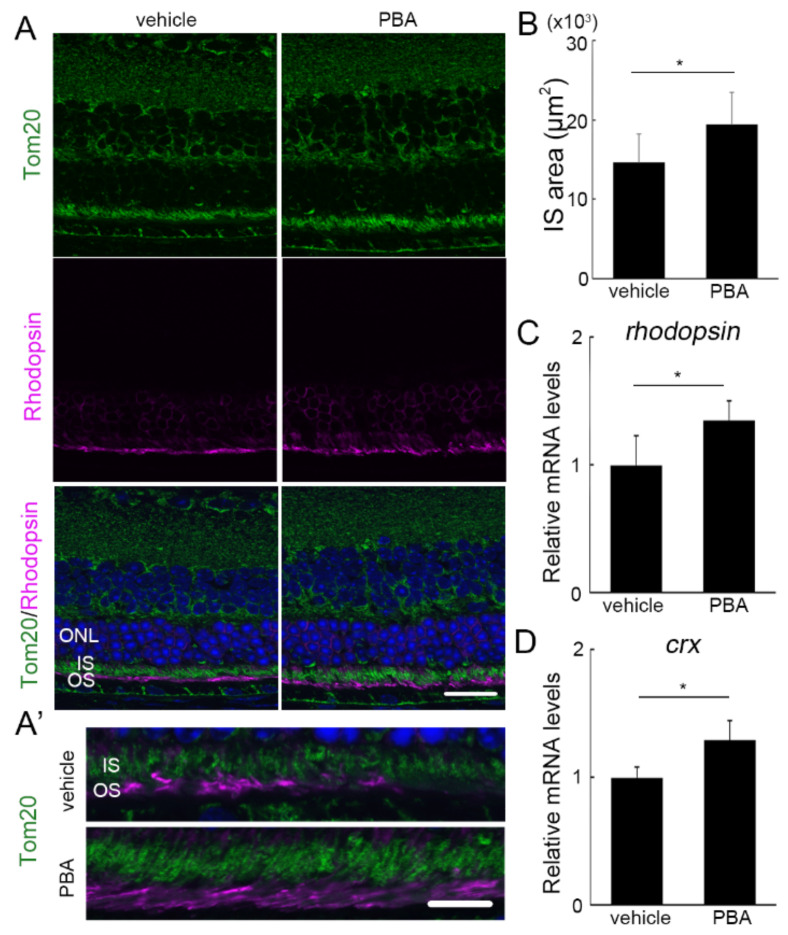
**Protective effect of PBA in the photoreceptors of P23H RP models detected before photoreceptor loss** (**A**,**A’**) Immunohistochemistry for Tom20 (green) and rhodopsin (pink) in the retina of P23H RP models at the age of 4 weeks with or without PBA treatment. Nuclei were counterstained with DAPI (blue). (**A**’) Magnified images of IS and OS. IS area (**B**), and mRNA levels of *rhodopsin* (**C**) and *crx* (**D**) in the retina were already different with or without PBA treatment. Data are shown as mean ± standard deviation. Respective *n* for P23H RP models treated with vehicle and PBA, (**B**) 4 and 5; (**C**) 5 and 4; (**D**) 10 and 10. ONL, outer nuclear layer; IS, inner segments; OS, outer segments. * *p* < 0.05. Scale bar, (**A**) 20 μm; (**B**) 10 μm.

At 4 weeks of age, when the PBA treatment had been continued for 2 weeks, there was no difference in the number of remaining photoreceptor cells between the PBA- and vehicle-treated groups in the P23H RP models (Figure 3A and Figure S2). However, the IS area, evaluated with Tom20 staining in IS, exhibited a difference between the groups with or without PBA treatment (Figure 3A,A’,B). The data suggested that mitochondrial damage may have been attenuated by PBA in the models. At that time, the mRNA levels of photoreceptor markers, *rhodopsin* (Figure 3C), and *crx*, a transcription factor upstream of rhodopsin (Figure 3D), were at significantly higher levels in the retina of PBA-treated P23H RP models, indicating that PBA treatment retained visual pigment expression in the rod photoreceptor cells compared with vehicle treatment.

### 3.4. PBA Induced ERAD and Mitochondrial Markers in the Retina of P23H RP Models

**Figure 4:**
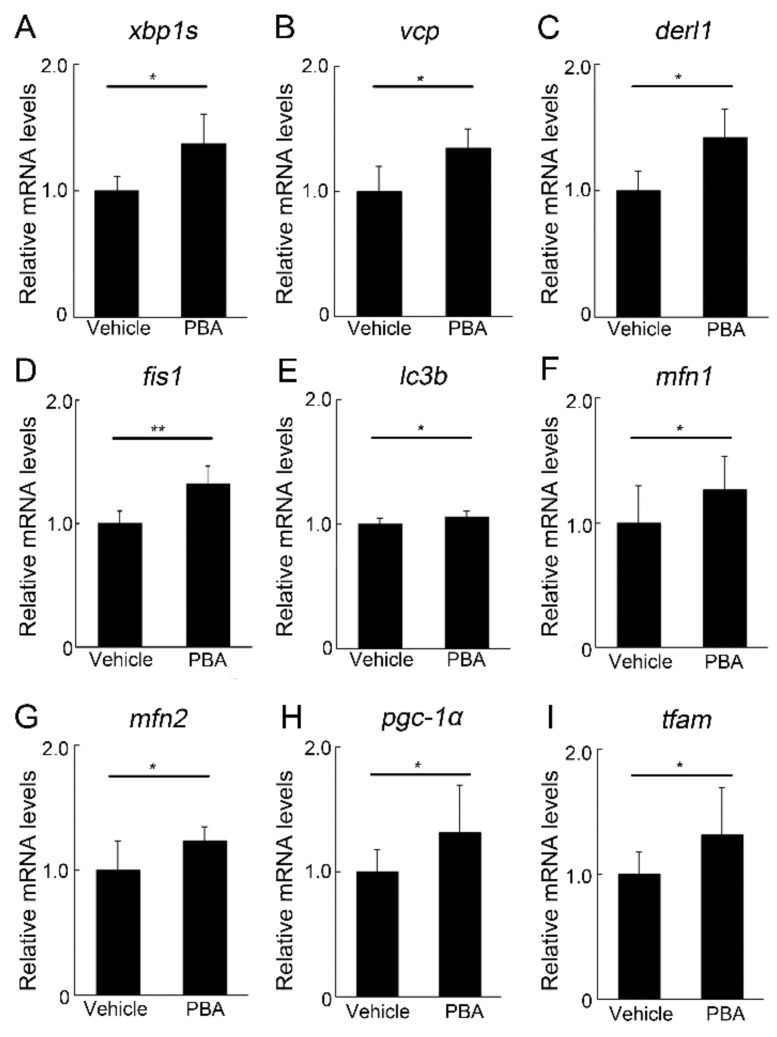
**Induction of ERAD and mitochondrial marker mRNAs by PBA in the retina of P23H RP models** (**A**–**I**) Real-time RT-PCR of the retinal samples obtained at the age of 4 weeks with or without PBA treatment. ERAD-related (**A**–**C**), mitochondrial kinetic (**D**,**F**,**G**) and autophagy (**E**) markers, *pgc-1α* (**H**), and *tfam* (**I**) were upregulated by PBA treatment. ERAD, ER-associated degradation. Data are shown as mean ± standard deviation. *n* for P23H RP models treated with vehicle and PBA, (**A**–**C**) 4–5; (**D**–**I**) 7–10. * *p* < 0.05, ** *p* < 0.01.

While P23H mutated rhodopsin causes ER stress [19,28,29,30,31,32,33], the ERAD system that disposes of abnormal proteins [16,17] is also induced in the models [19]. ERAD is activated by IRE1-related conversion of XBP1 to XBP1s, leading to induction of VCP (also known as Cdc48 or p97) interacting with Derlin 1 to transport the specific misfolded proteins from the ER to the cytosol utilizing its adenosine triphosphate (ATP) ase activity [34]. The misfolded protein delivered to the cytosol is processed to be degraded through the ubiquitin proteasome system (UPS) [16,17]. We found that PBA treatment upregulated *xbp1s* (Figure 4A), *vcp* (Figure 4B), and *derlin 1* (Figure 4C) in the retina of P23H RP models at the age of 4 weeks. These results suggested that PBA promoted ERAD induction, which could eliminate pathological P23H rhodopsin.

ER stress also affects mitochondrial quality control by regulating mitochondrial fission and fusion by VCP [34,35]; VCP induces fission to eliminate abnormal mitochondria through autophagy [35,36], whereas it induces degradation of fusion-related mitofusin proteins through UPS [34,35] to preserve cellular homeostasis [37]. The system is reported to be involved in neural plasticity and survival [38]. We found that PBA treatment increased the mRNA levels of the mitochondrial fission marker, *fis1* (Figure 4D), and an autophagy marker, *lc3b* (Figure 4E), in the retina of P23H RP models. The mRNA levels of fusion markers, *mfn1* (Figure 4F) and *mfn2* (Figure 4G), were also affected by PBA.

Mitochondrial biogenesis, which replaces damaged mitochondria with new and healthy ones [39], is affected during mitochondrial quality control [35]. We found that pgc-1α (Figure 4H), which regulates mitochondrial biogenesis [40], and *tfam* (Figure 4I), which represents mitochondrial DNA levels and is a transcription factor for inducing mitochondrial DNA encoding respiratory molecules [41], were both upregulated in the retina of P23H RP models treated with PBA, suggesting that mitochondrial biogenesis was activated by PBA treatment.

### 3.5. PBA Activated Oxidative Phosphorylation (OXPHOS)

**Figure 5:**
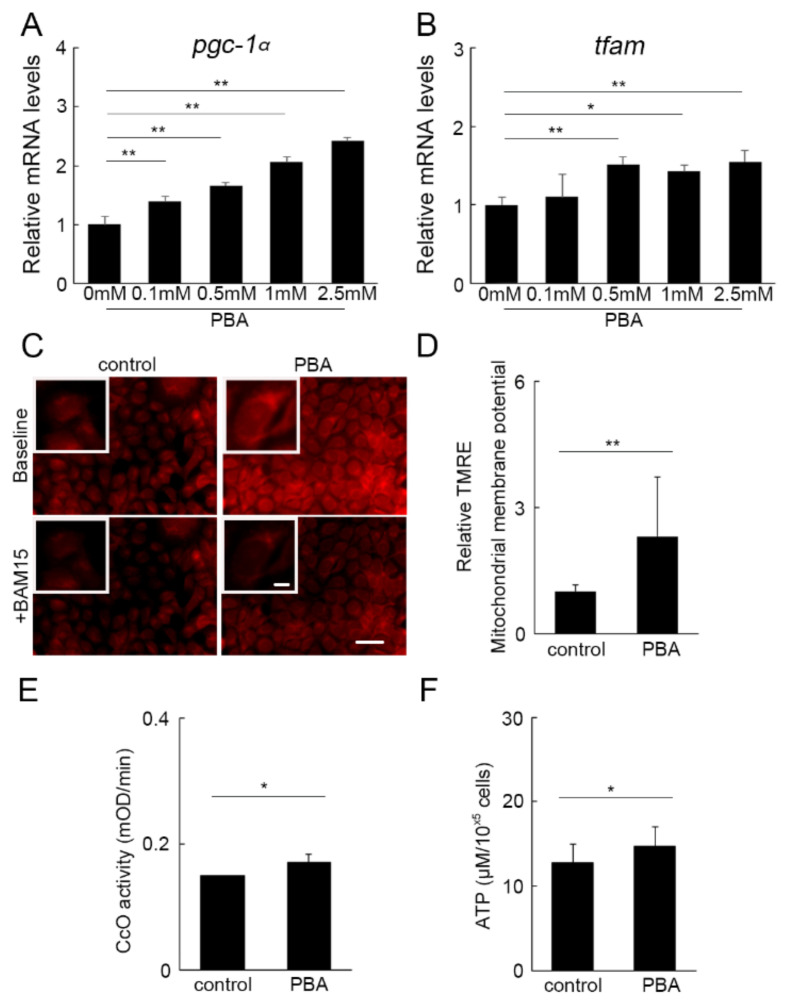
**OXPHOS activation by PBA** (**A**–**F**) HEK 293 cells incubated with or without PBA for 24 h. RT-PCR showed that *pgc-1α* (**A**) and *tfam* (**B**) increased by PBA in a dose-dependent manner. Mitochondrial membrane potential measured using TMRE, a fluorescent dye (**C**) showed that PBA increased the membrane potential (**C**,**D**). BAM15, a mitochondria protonophore uncoupler, was added to cancel the membrane potential, and the subtracted values of the fluorescence intensity representing the potential were shown in the graph (**D**). CoxIV (CcO) activity (**E**) and ATP levels (**F**) also increased by PBA. Data are shown as mean ± standard deviation. *n* for vehicle and PBA groups, (**A**–**E**) 3–4; (**F**) 13–14. OXPHOS, oxidative phosphorylation, CcO; cytochrome c oxidase. * *p* < 0.05, ** *p* < 0.01. Scale bars; 50 μm for the images in the large panels, and 10 μm for the magnified images in the insets.

As shown above, the mitochondrial-concentrated part of the photoreceptor, IS, was preserved by PBA, and the mitochondrial biogenesis marker was upregulated by PBA in vivo. Therefore, we further analyzed the potential effects of PBA on mitochondria using a HEK293 cell line, where PBA upregulated mRNA of *pgc-1α* (Figure 5A) and *tfam* (Figure 5B) in a dose-dependent manner. The mitochondrial membrane potential, which is indispensable for ATP synthesis and assessed using the subtraction method of the potential before and after administration of a protonophore uncoupler, BAM15, was increased in the cells treated by PBA (Figure 5C,D). Moreover, the activity of complex IV in the electron transport chain, also named cytochrome c oxidase (CcO), was increased by PBA (Figure 5E), and the ATP levels were increased by PBA (Figure 5F). These results indicated that PBA could promote mitochondrial function to increase ATP levels, which is required to eliminate abnormal unfolded proteins and for cytoprotection [21,42,43].

### 3.6. PBA Increased Histone Acetylation of the PGC-1α Promoter

**Figure 6:**
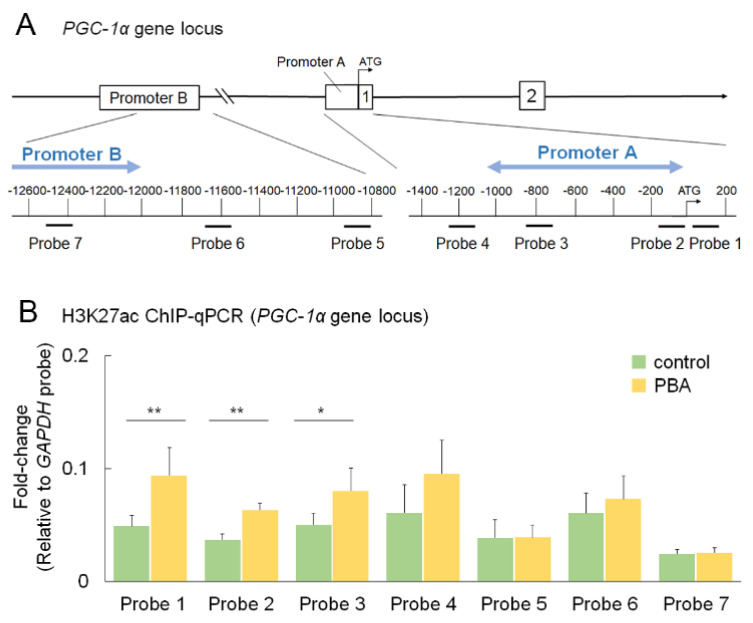
**Histone acetylation of PGC-1α promoter by PBA** (**A**) Schematic illustration of upstream of human PGC-1α gene including promoters A and B. (**B**) ChIP-qPCR showed that amplified DNA levels were significantly increased with probes 1, 2, and 3, which are involved in promoter A. Data are shown as mean ± standard deviation. Respective *n* for control and PBA; 5 and 6. * *p* < 0.05, ** *p* < 0.01.

We performed ChIP-qPCR assays at promoters A and B of the *PGC-1α* gene [44] using HEK293 cells to explore the mechanism of *pgc-1α* induction by PBA. We found that the acetylation of histone (H3K27ac) was increased by PBA at promoter A as shown by probes one, two, and three (Figure 6A,B), indicating that PBA can promote *pgc-1α* transcription through epigenetic modification, whereas promoter B of PGC-1α may not be involved in PBA-induced upregulation of *pgc-1α* (Figure 6B).

## 4. Discussion

Photoreceptor loss in the retina of P23H RP models was attenuated, and visual function was retained by constant administration of PBA. In the retina of P23H RP models, biomarkers for ERAD and mitochondrial quality control as well as mitochondrial biogenesis were upregulated. In vitro experiments showed that PBA induced *pgc-1α* and *tfam* mRNA and increased the mitochondrial membrane potential, CcO activity, and ATP levels. Histone acetylation of PGC-1α promoter A was increased by PBA.

The P23H mutant rhodopsin protein forms aggregates, causing ER stress [19,28,29,30,31,32,33], and is not delivered to OS but remains in IS, where ERs exist [45]. However, a previous report showed that metformin administration, which reduces aggregates of mutant rhodopsin and allows delivery of mutant rhodopsin to the OS, did not suppress but exacerbated photoreceptor death in P23H mutant models [46]. Moreover, CHOP, which is a death signal that is induced when the cells cannot compensate for the ER stress, was not involved in photoreceptor death in the P23H RP models; a CHOP knock-out background does not improve photoreceptor survival in the models [20,47]. In addition, when treated with a PERK inhibitor to suppress one of the ER-stress pathways, the visual function was exacerbated in a P23H transgenic rat model [48], and aggregates of mutant rhodopsin were increased in cells overexpressing P23H mutant rhodopsin [48]. This could be related to the fact that PERK can activate Nrf2, an antioxidative and protective transcription factor, in P23H mutants [49]. These results indicate that simple suppression of ER-stress signals is not an effective treatment in P23H RP models.

In contrast, we succeeded in demonstrating the neuroprotective effect of PBA, a chemical chaperon that is well-known as an ER-stress inhibitor [10,11] in the P23H RP models. In fact, PBA increased the ER stress-related molecule, *xbp1s*, which most likely increased the expression of its downstream molecules related to ERAD, such as *vcp* and *derlin1*. Thus, PBA may have promoted adaptive alteration by activating the ERAD pathway. XBP1s promote VCP-dependent translocation of unfolded proteins in an ATP-dependent manner to finally process the proteins to be degraded through UPS, which also consumes ATP [16,17]. A previous report has shown the reduction in the ratio of the insoluble protein and ER stress markers by PBA treatment [13], which could have been, at least in part, through activating ERAD, in addition to correcting the folding of misfolded and unfolded proteins [13].

Moreover, XBP1s-induced VCP also acts on mitochondrial quality control; it promotes fission and suppresses the fusion of mitochondria [16,17]. Upregulation of both *fis1* and *lc3b* in retinas treated with PBA in P23H RP models may have increased mitochondrial fission followed by mitophagy to eliminate unhealthy mitochondria through degradation [37]. This may have been advantageous to effectively respond to the increased energy demand required for ERAD. Moreover, activation of autophagy supports suppressing ER stress [50], which could have decreased energy demand. Meanwhile, autophagy itself may not be supportive of photoreceptor protection, according to previous reports of Xenopus P23H rhodopsin mutants [51]. Increased expression of the fusion marker, *mfn1,* and *mfn2* mRNA was most likely due to negative feedback related to the degradation of the fusion proteins by VCP [16,17]. *pgc-1α* and *tfam*, which represent mitochondrial function, and OXPHOS, for energy production [52] were also upregulated by PBA treatment. This may have resulted in preserving the IS area of the photoreceptors where mitochondria are distributed by PBA treatment. Previous reports have shown that metabolic failure was observed in the process of photoreceptor degeneration in rhodopsin-mutant Drosophila [53], and supplying sufficient energy contributes to photoreceptor survival in the retina under stress conditions [21,42,43,54]. Activation of adenosine 5′-monophosphate-activated protein kinase (AMPK) leading to energy supply by increasing ATP [21] or suppressing ATP degeneration [54,55] contributes to photoreceptor survival in mouse models. PBA may have had a role in mitochondrial quality control and effectively promoted the energy supply that was required for ERAD activation by inducing *pgc-1α*, a master regulator of mitochondrial biogenesis [56,57].

We demonstrated that PBA could increase promoter activation of the *PGC-1α* gene by histone acetylation of promoter A, which most likely upregulated OXPHOS and ATP production. A previous report showed that the MEF2-binding site of the *Pgc-1α* promoter could be deacetylated by HDAC5, one of the class IIa HDACs [58], leading to repression of *pgc-1α* transcriptional induction [59]. PBA is reported to be an inhibitor of class I and IIa HDAC [60], and our results showed that it increased the frequency of open chromatin at promoter A, where the MEF2-binding site is included. To the best of our knowledge, the current study firstly showed the epigenetic action of PBA on *PGC-1α*. Inhibition of excessively activated HDACs I/IIa in the degenerating retina of rd1 RP model mice, which have a loss-of-function mutation in the gene encoding for the β-subunit of rod photoreceptor cGMP phosphodiesterase-6, was reported to suppress photoreceptor death [61]; whether the effect involved *PGC-1α* upregulation would be an interesting point as a future study.

Because the P23H mutant rhodopsin protein is expressed in the rod photoreceptor cells, rod degeneration is the dominant change; however, the cone photoreceptors are also affected [20] and were also protected by PBA in the current study. The cone photoreceptors are protected by rod-derived cone viable factor (RdCVF) [62] through regulation of glucose metabolism [63] and oxidative stress [64], and administration of RdCVF was reported to rescue the cone photoreceptors in P23H rhodopsin-mutant rats [65]. PBA would have protected the cone photoreceptors by protecting the rod photoreceptors, and a direct pathway might have also been involved.

Because ER stress-mediated RP is also observed in the other mutations [66], whether PBA can be applicable to mutations other than P23H rhodopsin should also be studied in the future. Apart from RP, ER-stress-mediated pathogenesis is also reported in other fields, e.g., as a diabetic complication [67]. In addition, it will be interesting to study whether drug therapy with PBA can increase the therapeutic effect when used in combination with other treatments.

**Figure 7:**
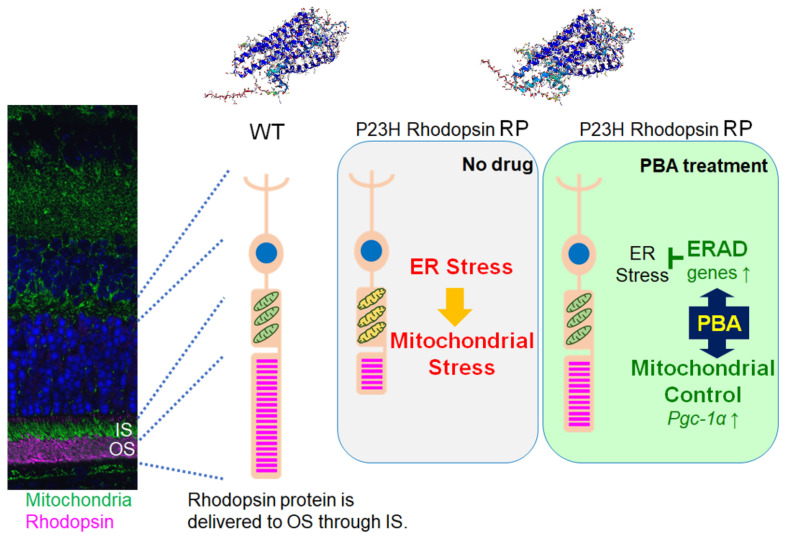
**Proposed roles of PBA in photoreceptor neuroprotection and visual protection** P23H rhodopsin mutant protein causes misfolding and ER stress, and may have damaged mitochondria, thus weaken photoreceptors and impaired visual function. In contrast, constant PBA administration induced ERAD, and moreover, *pgc-1α* most likely through epigenetic change and may have affected mitochondrial quality control leading to supply ATP required to run the ERAD system, which protected photoreceptors and visual function. Protein structures (alphafold2; https://alphafold.ebi.ac.uk/ (accessed 14 February 2022) are shown at the top (left, wild-type rhodopsin; right, P23H mutant rhodopsin). IS, inner segment (where ER and mitochondria are densely distributed); OS, outer segment (where rhodopsin is concentrated).

In summary, constant PBA administration attenuated photoreceptor death and rod and cone photoreceptor-related visual-function impairment in P23H RP models. The neuroprotective effect of PBA may have involved ERAD activation with the ATP supply required to operate the ERAD system and self-defense system, which was supported by mitochondrial quality control induced by PBA (Figure 7). The fact that PBA promoted acetylation of a *PGC-1α* promoter is informative for the future application of PBA in the clinical setting and for the possible adaptation of PBA treatment to the other medical fields, although further studies are required.

## Data Availability

All data generated or analyzed during this study are included in this published article and its Supplementary Information.

## Supplementary Materials

The following are available online at: https://www.mdpi.com/article/10.3390/cells11091497/s1, Figure S1: Spider graphs showing the number of ONL nuclei and IS area at each part of the retina. Figure S2: Number of photoreceptors in the P23H knock-in heterozygotes treated with PBA five times a week from 2 weeks until 4 weeks of age was comparable to those treated with vehicle, Table S1: Primers for RT-PCR, and Table S2: Primers for ChIP-qPCR.
