# Supplementary material for: Effects of Epigenetic Modification of PGC-1α by a Chemical Chaperon on Mitochondria Biogenesis and Visual Function in Retinitis Pigmentosa
Source: Cells. 2022 Apr 29;11(9):1497. doi: 10.3390/cells11091497 (PMC9099608; doi:10.3390/cells11091497)
Supplement: Supplementary file 1 [file cells-11-01497-s001.zip › cells-1617063 Supplementary Material.pdf]

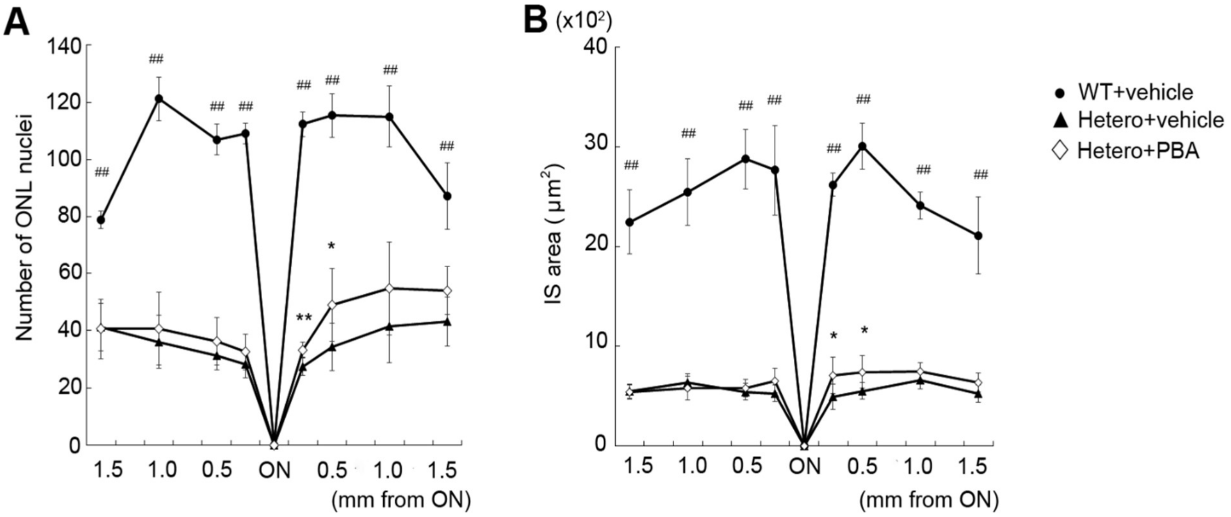


**Figure S1. Spider graphs showing the number of ONL nuclei and IS area at each part of the retina**

The number of ONL nuclei (A) and IS area (B) at each part of the retina were shown. Protective effects of PBA were observed in the superior retina as shown in the right side to the ON in the graphs (A, B). n for WT treated with vehicle, 3; P23H RP models treated with vehicle, 8–9; P23H treated with PBA, 8. ON, optic nerve. * $p < 0.05$, ** $p < 0.01$ for comparing P23H knock-in heter-ozygotes treated with vehicle and PBA, and ## $p <0.01$ for comparing WT and P23H knock-in heterozygotes treated with vehicle.

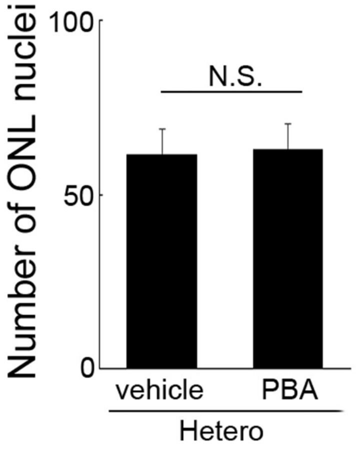


**Figure S2. Number of photoreceptors in the P23H knock-in heterozygotes treated with PBA five times a week from 2 weeks until 4 weeks of age was comparable to those treated with vehicle**

Data are shown as mean ± standard deviation. n = 6–8. p = 0.71. N.S., not significant.

**Table S1.** Primers for RT-PCR

| gene (mouse) | **Forward primer (5′–3′)** | **Reverse primer (3′–5′)** |
|---|---|---|
| *Gapdh* | AGGAGCGAGACCCCACTAAC | GATGACCCTTTTGGCTCCAC |
| *Rhodopsin* | AACTTCGGCCCCATCTTCA | CAGTGGATTCTTGCCGCAG |
| *Crx* | TGGAGGAGCTGGAGGCCCTGTTTGCCAAGAC | CCAAAGGATCTGTACAAACATCTGTAGAG |
| *xbp1s* | CTGAGTCCGCAGCAGGTG | TGCCCAAAAGGATATCAGACT |
| *Vcp* | AAGTCCCCAGTTGCCAAGGATG | AGCCGATGGATTTGTCTGCCTC |
| *Derl1* | CGCGATTTAAGGCCTGTTAC | GGTAGCCAGCGGTACAAAAA |
| *Fis1* | ATATGCCTGGTGCCTGGTTC | AGTCCCGCTGTTCCTCTTTG |
| *Mfn1* | GATGTCACCACAGAGCTGGA | AGAGCCGCTCATTCACCTTA |
| *Mfn2* | CCCCTCTCAAGCACTTTGTC | ACCTGCTCTTCCGTGGTAAC |
| *Pgc1a* | GATGAATACCGCAAAGAGCA | AGATTTACGGTGCATTCCT |
| *Tfam* | AGTCAGCTGATGGGTATGGAGAA | TGCTGAACGAGGTCTTTTTGG |
| *Lc3b* | Taqman probe; Mm00782868 | |

| gene (human) | **Forward primer (5′–3′)** | **Forward primer (5′–3′)** |
|---|---|---|
| *Gapdh* | CACCCACTCCTCCACCTTT | TCCACCACCCTGTTGCTGTAG |
| *Pgc1a* | GCACCGAAATTCTCCCTTG | GCCTCTCGTGCTGATATTCC |
| *Tfam* | AAGATTCCAAGAAGCTAAGGGTGA | CAGAGTCAGACAGATTTTTCCAGTTT |

**Table S2.** Primers for ChIP-qPCR

| | **Forward primer (5′–3′)** | **Reverse primer (3′–5′)** |
|---|---|---|
| promoter gapdh | ACTAGGCGCTCACTGTTCTC | GTTGACTCCGACCTTCACCT |
| promoter pgc-1α | | |
| probe 1 | TAATAGCATCTGAGGGAAGCGTC | GTGCAACCAGGACTCTGAGT |
| probe 2 | ACTCCAATCCACAGTGACACAG | GACAGGTGCCTTCAGTTCA |
| probe 3 | AGCTCCCGAAGAGTTGCTG | GAAGGAAGCTGAAAGGATGGG |
| probe 4 | AATGAGGGCTAATGCAGGTAGG | CCACTGTGTCCAGTACCTTGA |
| probe 5 | AATCCAGCCTCATACCAGTCC | CTTCACTTGCTGCTTCTGGTC |
| probe 6 | TGGGCAACAGAGTAAGACTCC | CCTCTCATTGTCCCAAGCTCAT |
| probe 7 | CCCAGCAATGCACAACTCC | GGAGAATGATTGCACAGCAT |
